# Supplementary material for: Causal interplay between lactose intolerance and gut microbiota: a combined bidirectional Mendelian randomization and in vivo validation study
Source: Front Nutr. 2026 Jun 1;13:1803337. doi: 10.3389/fnut.2026.1803337 (PMC13265576; doi:10.3389/fnut.2026.1803337)
Supplement: Supplementary file 1 [file Data_Sheet_1.zip › supplementary materials/Forward/leave one out cross validation/ebi-a-GCST90027633.finngen_R12_E4_LACTONAS.pdf]

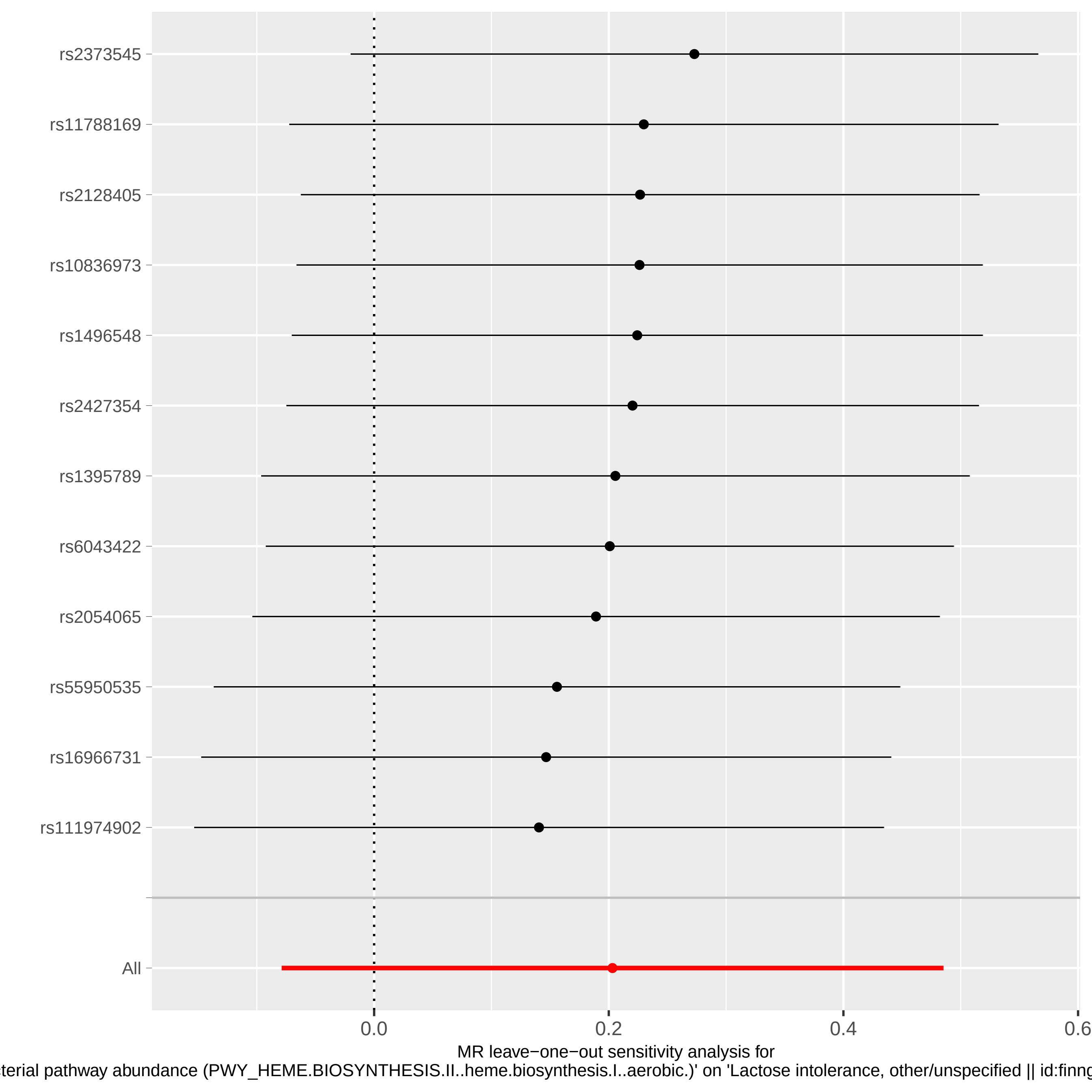
rs2373545
rs11788169
rs2128405
rs10836973
rs1496548
rs2427354
rs1395789
rs6043422
rs2054065
rs55950535
rs16966731
rs111974902
All
0.0
0.2
0.4
0.6
MR leave−one−out sensitivity analysis for
terial pathway abundance (PWY_HEME.BIOSYNTHESIS.II..heme.biosynthesis.I..aerobic.)' on 'Lactose intolerance, other/unspecified || id:finng
